# Retraction: Long noncoding RNA XIST is a prognostic factor in colorectal cancer and inhibits 5-fluorouracil-induced cell cytotoxicity through promoting thymidylate synthase expression

**DOI:** 10.18632/oncotarget.28897

**Published:** 2026-06-17

**Authors:** Yang Xiao, Usenko Alexander Yurievich, Smorzhevskyi Valentyn Yosypovych

**Affiliations:** ^1^Department of Surgery and Transplantology, P. L. Shupyk National Medical Academy of Postgraduate Education, Kyiv, Ukraine

**This article has been retracted:** Oncotarget has completed its investigation of this paper. The investigation found that multiple images in Figure 6B and Figure 4A were reproduced across several publications in different journals. Specifically:

- Figure 6B (HT29 cells, TS): This blot was found as Fibronectin in Figure 4E of article [1], as Bax in Figure 5C of article [2], as Bmi-1 in Figure 5D of article [3], and as Vimentin in Figure 1B of article [4].
- Figure 6B (HCT116 cells, TS): This blot was duplicated in article [5] as STAT1 (Figure 2B) and Bcl-2 (Figure 6B), and in article [6] as Bcl-2 (Figure 5C).
- Figure 6B (HCT116 cells, b-actin): This blot was reproduced as b-actin in Figure 5C of article [7] and as GAPDH in Figure 3G of article [8].
- Figure 4A: Three panels representing morphologic changes in 5FU resistant HT29 and HCT116 cells were found in Figure 1C of article [5] representing ESCC cells.

Due to these findings, the Editorial decision has been made to retract this paper. The authors were contacted multiple times to provide the explanations and original data, however, no response was received.

Original article: Oncotarget. 2017; 8:83171–83182. 83171-83182. https://doi.org/10.18632/oncotarget.20487

## References

[1] Liu T, Zhang J, Chai Z, Wang G, Cui N, Zhou B. Ginkgo biloba extract EGb 761-induced upregulation of LincRNA-p21 inhibits colorectal cancer metastasis by associating with EZH2. Oncotarget. 2017; 8:91614–27. 10.18632/oncotarget.21345. 29207671. Retraction in: Oncotarget. 2025; 16:310. DOI: 10.18632/oncotarget.28525 PMID: 40338158 PMC5710951

[2] Liu H, Zhou G, Fu X, Cui H, Pu G, Xiao Y, Sun W, Dong X, Zhang L, Cao S, Li G, Wu X, Yang X. Long noncoding RNA TUG1 is a diagnostic factor in lung adenocarcinoma and suppresses apoptosis via epigenetic silencing of BAX. Oncotarget. 2017; 8:101899–10. 10.18632/oncotarget.22058. 29254212. Retraction in: Oncotarget. 2024; 15:90. DOI: 10.18632/oncotarget.28462 PMID: 38329724 PMC5731922

[3] Zhou X, Natino D, Qin Z, Wang D, Tian Z, Cai X, Wang B, He X. Identification and functional characterization of circRNA-0008717 as an oncogene in osteosarcoma through sponging miR-203. Oncotarget. 2017; 9:22288–300. 10.18632/oncotarget.23466. 29854278 PMC5976464

[4] Dong H, Wang W, Chen R, Zhang Y, Zou K, Ye M, He X, Zhang F, Han J. Exosome-mediated transfer of lncRNA-SNHG14 promotes trastuzumab chemoresistance in breast cancer. Int J Oncol. 2018; 53:1013–26. 10.3892/ijo.2018.4467. 30015837. Retraction in: Int J Oncol. 2022; 61:92. DOI: 10.3892/ijo.2022.5382 PMID: 35686548 PMC6065402

[5] Kang M, Ren M, Li Y, Fu Y, Deng M, Li C. Exosome-mediated transfer of lncRNA PART1 induces gefitinib resistance in esophageal squamous cell carcinoma via functioning as a competing endogenous RNA. J Exp Clin Cancer Res. 2018; 37:171. 10.1186/s13046-018-0845-9. 30049286. Retraction in: J Exp Clin Cancer Res. 2023; 42:82. DOI: 10.1186/s13046-023-02648-7 PMID: 37024914 PMC6063009

[6] Dai W, Mu L, Cui Y, Li Y, Chen P, Xie H, Wang X. Berberine Promotes Apoptosis of Colorectal Cancer via Regulation of the Long Non-Coding RNA (lncRNA) Cancer Susceptibility Candidate 2 (CASC2)/AU-Binding Factor 1 (AUF1)/B-Cell CLL/Lymphoma 2 (Bcl-2) Axis. Med Sci Monit. 2019; 25:730–38. 10.12659/MSM.912082. 30681073 PMC6357823

[7] Li Z, Xu W, Ren X, Xu J, Chen J. Puerarin promotes DUSP1 expression by regulating miR-133a-3p in breast cancer. Mol Med Rep. 2019; 19:205–12. 10.3892/mmr.2018.9682. 30483784 PMC6297792

[8] Huang L, Wang ZY, Pan DD. Penicillin-binding protein 1A mutation-positive Helicobacter pylori promotes epithelial-mesenchymal transition in gastric cancer via the suppression of microRNA-134. Int J Oncol. 2019; 54:916–28. 10.3892/ijo.2018.4665. 30569124 PMC6365042

